# Editorial: Further findings in the role of inflammation in the etiology and treatment of schizophrenia

**DOI:** 10.3389/fpsyt.2024.1349568

**Published:** 2024-03-21

**Authors:** Massimo Tusconi, Serdar M. Dursun

**Affiliations:** ^1^ Section of Psychiatry, Department of Medical Sciences and Public Health, University of Cagliari, Cagliari, Italy; ^2^ Neurochemical Research Unit, Department of Psychiatry, University of Alberta, Edmonton, AB, Canada

**Keywords:** inflammation, biomarkers, neurobiology, neuroimaging, psychiatric genetics

Current biomedical knowledge makes it possible to ascertain how brain function represented by thought is subject to a series of changes in a pathological sense as the condition of anatomical microstructures in the brain changes and neurophysiology changes in response to environmental stimuli (1). Increasing evidence has pointed to the implication of inflammation in schizophrenia, and patients with diagnoses afferent to its spectrum may have increased pro-inflammatory markers (2) and a higher prevalence of inflammatory diseases. In addition, genetic and epigenetic studies have highlighted the role of immune and endocrinological systems in schizophrenia (3), and some clinical trials have detected antipsychotic effects of anti-inflammatory drugs (4). In light of these considerations, the Research Topic aimed to evaluate various molecular, biological, genetic, and neuroimaging aspects due to the consequences of inflammation to identify risk factors, predictors, and possible protective factors or specific treatment methods.

Regarding routine immunological laboratory parameters, Skalniak et al. highlight how their values measured at admission may act to improve positive symptoms in schizophrenia after treatment. From the data analysis, the authors repeatedly highlight the altered C-reactive protein (CRP) levels in patients with schizophrenia compared with healthy controls. According to further psychometric evaluation using the PANSS scale, correlation with inflammation parameters is present in positive PANSS scales on admission; these parameters also decline significantly after drug treatment. The authors found that for PANSS subscales representing arousal and disorganization of thought processes, the immunological parameters C4 and CRP, respectively, parametrically modify the outcome of drug treatment. Further assessing negative symptoms, fT3, glucose and creatinine levels appear to be substantial modifiers, while creatinine affects the arousal subscales and HDL affects the subscales describing negative emotions. In their study on schizophrenia and cellular senescence candidate gene screening, machine learning, diagnostic models, and drug prediction, Feng et al. demonstrate through KEGG analysis the connection between Epstein-Barr virus (EBV) infection and schizophrenia-related genes; continuing in an overlap between genes related to cellular senescence and those correlated with schizophrenia, they point out a further link of these genes to EBV infection, emphasizing the implicit link between schizophrenia and immunity. Using an analysis of immune infiltration in their work, they place emphasis on the differences in levels of follicular t-helper cells between schizophrenia and healthy controls, creating a diagnostic model based on genes concerning senescence, particularly genes IRF3, IRF7, MYLK, ID1, SFN, and KDM5B. Each of the analyzed genes exerts its action in a culminating manner both at the level of cellular aging and the genesis and clinical course of schizophrenia, highlighting how a more in-depth description of them may help to clarify yet unaccounted-for aspects of the pathogenesis process. In their study regarding changes in inflammatory markers after a 12-week exercise program in individuals with schizophrenia, Bigseth et al. report how in an evaluation regarding IL-6 and TNF they showed no significant differences between the groups (high-intensity interval training or video gaming) studied, but reported a marked increase in these markers from baseline to post-intervention after 3 months. Through the analysis and study of three different models with non-similar effects for each of the two inflammatory markers, the authors longitudinally showed variations regarding IL-6; in particular, they reported how an elevated level of this marker at baseline was associated significantly with cardiorespiratory fitness (CRF) during the intervention. In an additional cross-sectional study regarding inflammatory markers in outpatients diagnosed with schizophrenia who regularly use clozapine, Cordova et al. used the Brief Psychiatric Rating Scale (BPRS) to assess the severity of psychiatric symptoms. Based on their analysis, clozapine users showed significantly higher neutrophil counts than nonusers of clozapine. Patients on amitriptyline treatment or concomitant therapy by additional antipsychotic drugs had higher BPRS scale scores than patients not treated with the same drugs. The authors emphasized by multivariate analysis the link between amitriptyline and reduction in the systemic immune inflammation index (SII) and leukocyte ratios (NLR, MLR, and LPR), corroborating previous research on an effect of tricyclic antidepressants in anti-inflammatory terms. These findings suggest that it is relevant to evaluate these markers as potential predictive keys to clinical response to clozapine treatment.

Chaves et al., in their opinion article, discuss the possibility of the existence of a link between neuroinflammation and schizophrenia. In particular, they advance the hypothesis that inflammatory molecules, including cytokines and chemokines (TNF-α, IL-1β, IL-6), when activated prenatally can enter the placenta to the detriment of general fetal conditions, particularly brain development, by altering trajectories concerning neurodevelopment. It is also highlighted that the vulnerability-stress-inflammation model of schizophrenia has been analyzed in multiple epidemiological and genetic studies, thus departing from the traditional model of stress disease and neural predisposition. The authors analyze the relevance of neuroprogression and potential biomarkers for the diagnosis of schizophrenia and its treatment, emphasizing how identification of potential inflammation-related biomarkers can lead to precision diagnosis and tailoring of treatments.

Schizophrenia is a severe chronic mental disorder characterized by a great multiplicity of concurrences and by environmental, genetic and epigenetic influences involving numerous biomarkers (5). The involvement of such a broad procession of factors means that schizophrenic pathology has comorbidity with a large number of pathologies and is overlapping in treatment and diagnostic processes with several specialties in medicine. Careful evaluation and in-depth study of biological markers related to inflammatory states related to psychotic spectrum disorders may lead to the identification of new parameters of care for patients, ascertainment of the psychopathological mechanisms, increased opposition to stigma, and achievement of better outcomes.

## Author contributions

MT: Writing – review & editing, Writing – original draft, Conceptualization. SD: Writing – review & editing, Writing – original draft, Conceptualization.
